# That Law

**Published:** 1874-10

**Authors:** 


### The Bistoury.

ELMIRA, N. Y., OCT., 1874.

The BISTOURY is published Quarterly, upon the 1st of April, July, October and January, at Fifty Cents a year, in advance.

### THAT LAW.

Our last legislature, in its infinite wisdom, passed a law which at once received the approval of the Governor, kindly selecting for the commonwealth and every individual thereof, such men as to them (the gentlemen of the legislature), seemed best qualified to act in the capacity of our respective family physicians. Or, to make the statement more terse and concise, the legislature propose to select our medical attendants for us. The law requires that "every practitioner of medicine or surgery in this State, except licentiates or graduates of some medical society or chartered school, shall obtain a certificate from the censors of some one of the several medical societies of this State," etc. That is all very well, and very kind of our respected legislature; we believe with them, that those best qualified to practice medicine or surgery are those who have been properly and thoroughly educated in the profession. But do we always find them so qualified in the "several medical societies" referred to ?—Scarcely! We are certified that at least two individuals from this county, of very slender intelligence and less medical knowledge, (indeed, pretending to none save that obtained through the spirit of some defunct old doctor, who, if his spirit-land-prescriptions are a sample of what his ability must have been while upon this mundane sphere, he certainly never could have been admitted by reason of his competency, even to the Chemung County Medical Society), were, in consideration of a small fee, given certificates from "one of the several medical societies" before named, in which they were declared competent to practice medicine or surgery. In view of these facts, we fail to see any "protection to the people, from the practice of incompetent persons," and we doubt whether death would be any more acceptable from a licensed than from a non-licensed quack;—doubtless we should be quite as dead under either practice.

Our private opinion is that, if the regularly educated profession are unable to exhibit the superiority of their claims to public confidence and patronage, through the conspicuity of their skill, that no amount of legislation for the suppression of quackery will boost them into that much coveted position. And, further, it is our belief, that the more we try to "suppress quackery" through legislation, the more popular it will become, for it is contrary to the average American's idea of independence to have the right to select his own physician questioned, or in the least interfered with; and, while we would pity the credulence of any one stupid enough to desire the services of an ignorant "faith doctor" to attend him in his illness, yet would we accord to him the blessed privilege of being rubbed and purged by him if he so elects,—and who shall say him nay?

